# Individuals With Weaker Antibody Responses After Booster Immunization Are Prone to Omicron Breakthrough Infections

**DOI:** 10.3389/fimmu.2022.907343

**Published:** 2022-06-23

**Authors:** Birte Möhlendick, Ieva Čiučiulkaitė, Carina Elsner, Olympia E. Anastasiou, Mirko Trilling, Bernd Wagner, Denise Zwanziger, Karl-Heinz Jöckel, Ulf Dittmer, Winfried Siffert

**Affiliations:** ^1^ Institute of Pharmacogenetics, University Hospital Essen, University of Duisburg-Essen, Essen, Germany; ^2^ Institute for Virology, University Hospital Essen, University of Duisburg-Essen, Essen, Germany; ^3^ Department of Clinical Chemistry and Laboratory Medicine, University-Hospital Essen, University of Duisburg-Essen, Essen, Germany; ^4^ Department of Endocrinology, Diabetes and Metabolism and Division of Laboratory Research, University-Hospital Essen, University of Duisburg-Essen, Essen, Germany; ^5^ Institute of Medical Informatics, Biometry and Epidemiology, University of Duisburg-Essen, Essen, Germany

**Keywords:** SARS-CoV-2, booster vaccination, breakthrough infection, COVID-19, humoral immune response, neutralization, anti-spike antibodies

## Abstract

**Background:**

Despite the high level of protection against severe COVID-19 provided by the currently available vaccines some breakthrough infections occur. Until now, there is no information whether a potential risk of a breakthrough infection can be inferred from the level of antibodies after booster vaccination.

**Methods:**

Levels of binding antibodies and neutralization capacity after the first, one and six month after the second, and one month after the third (booster) vaccination against COVID-19 were measured in serum samples from 1391 healthcare workers at the University Hospital Essen. Demographics, vaccination scheme, pre-infection antibody titers and neutralization capacity were compared between individuals with and without breakthrough infections.

**Results:**

The risk of developing an Omicron breakthrough infection was independent of vaccination scheme, sex, body mass index, smoking status or pre-existing conditions. In participants with low pre-infection anti-spike antibodies (≤ 2641.0 BAU/ml) and weaker neutralization capacity (≤ 65.9%) against Omicron one month after the booster vaccination the risk for developing an Omicron infection was 10-fold increased (*P* = 0.001; 95% confidence interval, 2.36 - 47.55).

**Conclusion:**

Routine testing of anti-SARS-CoV-2 IgG antibodies and surrogate virus neutralization can quantify vaccine-induced humoral immune response and may help to identify subjects who are at risk for a breakthrough infection. The establishment of thresholds for SARS-CoV-2 IgG antibody levels identifying “non”-, “low” and “high”-responders may be used as an indication for re-vaccination.

## 1 Introduction

Despite the undeniable success of anti-Coronavirus disease 2019 (COVID-19) vaccines, some breakthrough infections occur. So far, the predisposing factors remain largely elusive. The lack of knowledge is based on three factors: (I) Retrospective surveillance studies lack clinical specimens predating the infection event. (II) Post-infection immune profiling is necessarily confounded by anamnestic immune responses. (III) Breakthrough infections are rare events. Thus, only well-powered prospective studies enroll sufficient participants to allow for a stratification according to the occurrence of breakthrough infections and a look-back assessment of pre-infection immunity. Here we report, to our knowledge for the first time, on such humoral immune responses being present in boostered vaccinees prior to severe acute respiratory syndrome coronavirus 2 (SARS-CoV-2) breakthrough infection with the Omicron variant.

Only two days after its description, the variant B.1.1.529 was declared as a variant of concern and designated with the Greek letter Omicron on November 26, 2021 (1). Omicron is highly transmissible, able to evade the immune system and currently approved vaccines are less effective against this variant (2–6).

The increased number of breakthrough infections observed during the Omicron wave allowed us to analyze pre-infection immune responses in a comprehensive longitudinal monocentric observational study cohort. Also, we were able to determine a threshold for anti-spike antibody levels and neutralization capacity at which the risk for a breakthrough infection significantly increases.

## 2 Methods

### 2.1 Study Cohort

Since April 2021, we have recruited 2526 healthcare workers at the University Hospital Essen (Essen, Germany) as participants for a comprehensive study on immune responses to vaccines against COVID-19.

Up to now 1391 participants have received their third SARS-CoV-2 vaccination (booster) with an mRNA vaccine after a first and a second vaccination with either mRNA (BNT162b2, BioNTech SE or mRNA-1273, Moderna Inc.), an adenoviral vector vaccine (AZD1222, AstraZeneca), or a combination of both. All individuals were vaccinated in accordance with recommendations of the national vaccine commission (STIKO). Study participants had to self-administer rapid SARS-CoV-2 nucleocapsid protein antigen tests at least twice a week since December 1, 2021. All subjects completed questionnaires at regular intervals delivering information on demographics, general health, and any known SARS-CoV-2 infection including symptoms and course of disease.

Between November 29, 2021, and March 5, 2022, 102 (7.3%) participants self-reported a SARS-CoV-2 infection. All infections were confirmed by real-time reverse transcription-PCR (RT-PCR). In 16 individuals (15.7%), an infection with the Omicron variant (B.1.1.529; BA.1 or BA.1.1) was confirmed by sequencing. Except for one case which occurred before the last week of December 2021, all other cases were observed when the Omicron variant was already predominantly or exclusively detected in healthcare workers and patients at the University Hospital Essen (**Supplementary Figure 1**). Our observations are consistent with the data reported by the Robert Koch Institute for Germany during this period (7). Thus, it is very unlikely, that variants other than Omicron contributed significantly to herein analyzed breakthrough infections. Association of age, body mass index (BMI, kg/m^2^) to breakthrough infection were estimated by Mann-Whitney test. *P*-value, odds ratio (OR) and 95% confidence interval (CI) were calculated for association of sex, pre-existing conditions, smoking status, or vaccination scheme to SARS-CoV-2 vaccination breakthrough infection.

### 2.2 Laboratory and Statistical Methods

#### 2.2.1 SARS-CoV-2 Spike Protein Immunoassay

The determination of anti-spike SARS-CoV-2 antibody concentrations was performed at specific time points (**Supplementary Figure 2**) using SARS-CoV-2 S1 RBD IgG/sCOVG test on Siemens Atellica^®^ IM System (Siemens Healthcare GmbH, Erlangen, Germany) according to the manufacturers’ instructions. Results of anti-spike antibody levels were given in binding antibody units per milliliter serum (BAU/ml). The detection limit for positivity was 21.8 BAU/ml.

Distribution of data was assessed by Shapiro-Wilk test prior association analysis. Data were not normally distributed (*P* < 0.0001). Association of anti-spike antibody levels with vaccination breakthrough was estimated by Mann-Whitney test. To determine a threshold above which the risk for a breakthrough infection significantly increases, the frequency distribution of anti-spike antibody levels at the 25% percentile (2816.0 BAU/ml) of all study participants one month after booster vaccination was analyzed and *P*-value by Fisher’s exact test as well as OR and 95% CI were calculated. Sensitivity and specificity of the selected cut-off were estimated by ROC analysis (sensitivity = 0.8; specificity = 0.6).

#### 2.2.2 SARS-CoV-2 Nucleocapsid Protein Immunoassay

All samples were also analyzed at the same time points (**Supplementary Figure 2**) for SARS-CoV-2 IgG antibodies against the nucleocapsid protein on the Architect i2000SR system (CoV-2 IgG, Abbott Diagnostics, IL, USA) to detect participants with previous SARS-CoV-2 infection. An index of ≥ 1.4 specimen calibrator (s/c) was considered as positive for a previous infection.

#### 2.2.3 SARS-CoV-2 Surrogate Neutralization Test (sVNT)

To detect circulating neutralizing antibody against SARS-CoV-2 which block the interaction between wild type (WT)- or Omicron-receptor binding domain (RBD; Accession #P0DTC2) we used a SARS-COV-2 surrogate virus neutralization test (sVNT; GenScript Biotech, Leiden, Netherlands). sVNTs have a high sensitivity and specificity and show a good correlation to conventional plaque reduction neutralization tests (8, 9) and, thus, can be used as a substitute test for cell-based neutralization assays.

All available samples of infected individuals (N = 62) one month after booster vaccination were analyzed and compared with a non-infected cohort (N = 53) matched in age, sex and vaccination scheme. Association of anti-spike antibody titers to breakthrough infection as seen in the complete cohort could be confirmed in this selected cohort (**Supplementary Figure 4**, 3477.0 BAU/ml vs 6935.0 BAU/ml, *P* < 0.0001).

Samples were diluted 1:576 for WT-RBD and 1:9 for Omicron-RBD and then incubated with the horseradish-peroxidase (HRP)-conjugated WT- or Omicron-RBD. The mixture was added to a capture plate coated with human ACE2 receptor protein (hACE2). Circulating neutralization antibodies which bound to the HRP-RBD complexes remained in the supernatant, whereas unbound WT- or Omicron-HRP-RBD were captured on the plate. After washing, 3,3’,5,5’-Tetramethylbenzidine (TMB) solution was added to the plate as a substrate for HRP. After the reaction was quenched by adding a stop solution, absorbance at 450 nm was measured on a microplate reader (FLUOstar Omega, BMG Labtech, Ortenberg, Germany). The absorbance of the sample is inversely correlated to the neutralization capacity of the sample. The inhibition rates (%) as an expression of the neutralization capacity for WT- and Omicron-RBD of the sample was calculated against negative control, respectively.

Distribution of data was assessed by Shapiro-Wilk test prior association analysis. Data were not normally distributed (*P* < 0.0001). Association of inhibition rate against Omicron-RBD and breakthrough infection risk was calculated by Mann-Whitney test. To determine a threshold above which the risk for a breakthrough infection significantly increases we analyzed the frequency distribution of inhibition rates at the 25% percentile (65.9%) in the breakthrough infection cohort compared to the matched controls one month after booster vaccination and calculated *P*-value by Fisher’s exact test, OR and 95% CI. Combined risk estimation was performed with cases and matched controls of the breakthrough infection cohort. Frequency distributions at the 25% percentiles of antibody titers (2641.0 BAU/ml, sensitivity = 1.0; specificity = 0.7 as estimated by ROC analysis) plus inhibition rates against Omicron-RBD (65.9%, sensitivity = 0.9; specificity = 0.7 as estimated by ROC analysis) were analyzed to determine a threshold above which breakthrough infection risk increases significantly.

## 3 Results

Between November 29, 2021, and March 5, 2022, 102 (7.3%) participants reported an RT-PCR-confirmed SARS-CoV-2 infection, whereas 1289 (92.7%) participants remained uninfected. On average, breakthrough infections occurred after 52 days (10 - 127) after the booster vaccination. With the exception of a slightly but significantly younger age (37 vs. 41 years, *P* = 0.004) SARS-CoV-2-infected subjects did not differ regarding vaccination scheme, sex, BMI, smoking status, or any other pre-existing conditions from the non-infected subjects (**Supplementary Table 1**).

The source of infection remained elusive for 28 subjects (27.4%). The majority of individuals reported that they had been infected in the familial or domestic environment (53.0%). In 27.0% of these cases, the infection could be traced back to other SARS-CoV-2-positive household members, such as the partner or roommates, and in 26.0% of the cases to a contact with their SARS-CoV-2-positive child. Eleven percent of the participants reported being infected while traveling or sporting activity. Only 8.0% of the subjects self-reported being infected at work.

All infections were described as a mild to moderate ″cold-like″ illness without a need for hospitalization. On average, symptoms lasted for six days (0 - 22 days), with rhinitis (53.9%), sore throat (52.9%), headache or cough (both 45.1%), and fatigue (34.3%) being the most prevalent symptoms. Other symptoms typically associated with COVID-19 such as fever (21.6%), dyspnea (15.7%), dysosmia (9.8%), and dysgeusia (6.9%) were rarely observed. Ten individuals (9.8%) remained asymptomatic.

We stratified our cohort according to the occurrence of an Omicron breakthrough infection, and then applied a look-back assessment to compare pre-infection antibody titers at various time points after vaccination but prior to infection (**Supplementary Figure 2**). Anti-spike antibody titers were indistinguishable between individuals with and without a later Omicron breakthrough infection after the first as well as one and six month after the second vaccination (**Supplementary Figure 3**).

**Figure 1:**
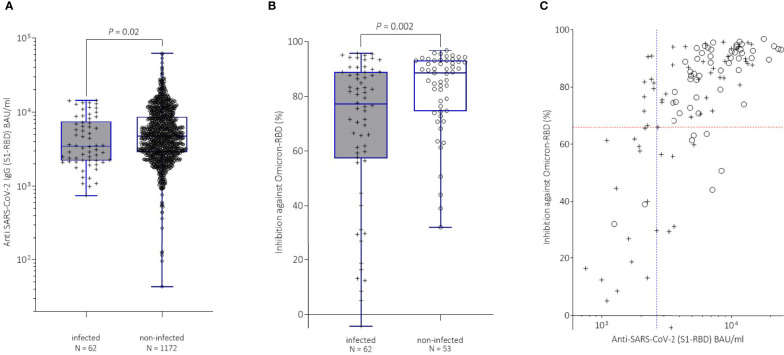
**(A)** Comparison of anti-spike antibody titers in study participants, with (+, grey boxplots) or without (o, white boxplots) breakthrough infection after the third vaccination. Antibody levels were determined one month after the third vaccination (booster). Median values between infected (3477.0 BAU/ml) and non-infected (4733.0 BAU/ml) individuals differed significantly (Mann-Whitney test; *P* = 0.02). **(B)** Comparison of neutralization capacity as measured by inhibition (%) against Omicron in the study participants with breakthrough infection (+, grey boxplots) with matched non-infected controls (o, white boxplots) after booster vaccination. Median inhibition rates between infected (77.1 %) and non-infected (88.5%) individuals differed significantly (Mann-Whitney test; *P* = 0.002). **(C)** Comparison of anti-spike antibody levels (BAU/ml) with inhibition rates (%) against Omicron-RBD in breakthrough infection cohort (+) and matched non-infected controls (o) by. The red dashed line indicates the 25% percentile (65.9 %) of inhibition rate. The blue dashed line denotes the 25%percentile (2641.0 BAU/ml) of anti-spike antibody level.

In clear contrast we observed a significant difference in anti-spike antibody levels one month after the booster vaccination between subjects with and without breakthrough infection before they became actually infected (3477.0 BAU/ml vs 4733.0 BAU/ml, *P* = 0.02; **Figure 1A**).

Study participants with an anti-spike antibody level of 2816.0 BAU/ml or less had a 2-fold increased risk for a breakthrough infection compared to individuals with antibody levels exceeding this cut-off (OR: 2.12, 95% CI: 1.24 - 3.58, *P* = 0.01).

Remarkably, inhibition rates against Omicron in the sVNT were also significantly lower in infected individuals prior to infection (77.1% vs 88.5%, *P* = 0.002; **Figure 1B**). Subjects with inhibition rates of 65.9% or less had a 3.6-fold increased risk for a breakthrough infection compared to SARS-CoV-2-negative study participants (OR: 3.61, 95% CI: 1.42 - 9.01, *P* = 0.01).

Subsequently, we combined the inhibition rates obtained in the sVNT together with anti-spike antibody levels of both infected and non-infected individuals (**Figure 1C**) and estimated the risk for breakthrough infections at low anti-spike binding titers and sVNT inhibition rates. Individuals with anti-spike antibody titers of 2641.0 BAU/ml or less plus a sVNT inhibition against Omicron of 65.9% or less showed a 10-fold increased risk for breakthrough infection compared with individuals with titers above these thresholds (OR: 10.4, 95% CI: 2.36 - 47.55).

## 4 Discussion

To the best of our knowledge, this is the first comprehensive study reporting lower antibody levels and a diminished neutralization capacity in individuals prior to a SARS-CoV-2 breakthrough infection with the Omicron variant compared to non-infected individuals based on a cohort of uniformly boostered vaccinees. Our data suggest that routine anti-SARS-CoV-2 IgG antibody determinations in combination with sVNT can identify subjects who are at a higher risk for a breakthrough infection.

Although a weaker immune response upon COVID-19 vaccination has been described for particular groups, like elderly or immune-compromised individuals (10–13), we observed differences in immune response independent from these factors. Genetic variations might explain some of the differences in the strength of innate immune response (14).

Higher humoral immune responses as determined by antispike-antibody determinations and sVNT tests have been reported for boostered vaccinees by several studies (15–20). Additionally, a higher immune response in boostered vaccinees compared to individuals, who only have been vaccinated twice, against the Omicron variant, which nevertheless is weaker than against the wild type virus, has been reported as well (21, 22). However, no thresholds for a higher probability of SARS-CoV-2 breakthrough infection have been established based on other comprehensive longitudinal studies so far.

Up to now, testing of anti-SARS-CoV-2 antibodies or neutralization capacity is not routinely performed after vaccination and, thus, “non”- or “low”-responders who might require a re-vaccination remain unidentified. Otherwise, the interval to re-vaccination in individuals with “high” response might be extended. Therefore, further studies are urgently needed to set a threshold for “non”, “low” and high” vaccination response to better plan vaccination strategies.

## Data Availability Statement

The original contributions presented in the study are included in the article/**Supplementary Material**. Further inquiries can be directed to the corresponding author.

## Ethics Statement

The studies involving human participants were reviewed and approved by Ethics Committee of the Medical Faculty of the University of Duisburg-Essen (21-10005-BO). The patients/participants provided their written informed consent to participate in this study.

## Author Contributions

BM, WS, and K-HJ conceptualized and designed the study. BM obtained ethics approval. BM and IC obtained informed consent, demographic data, and samples from the participants, organized logistics and performed laboratory analyses. Additionally, CE, OA, MT, BW, and DZ performed laboratory investigations. BM performed statistical analyses. BM, UD, and WS drafted and wrote the manuscript which was reviewed by all authors. All authors had full access to the data and approved submission of this article. BM and WS had the final responsibility to submit for publication.

## Funding

This work was supported by the Ministry of Culture and Science of North Rhine-Westphalia; VIRus Alliance NRW). The funder of the study had no role in study design, data collection, data analysis, data interpretation, writing of the report, or in decision of submitting the paper for publication. We acknowledge support by the Open Access Publication Fund of the University of Duisburg-Essen.

## Conflict of Interest

The authors declare that the research was conducted in the absence of any commercial or financial relationships that could be construed as a potential conflict of interest.

## Publisher’s Note

All claims expressed in this article are solely those of the authors and do not necessarily represent those of their affiliated organizations, or those of the publisher, the editors and the reviewers. Any product that may be evaluated in this article, or claim that may be made by its manufacturer, is not guaranteed or endorsed by the publisher.
