# Supplementary material for: Individuals With Weaker Antibody Responses After Booster Immunization Are Prone to Omicron Breakthrough Infections
Source: Front Immunol. 2022 Jun 23;13:907343. doi: 10.3389/fimmu.2022.907343 (PMC9260040; doi:10.3389/fimmu.2022.907343)
Supplement: Supplementary file 1 [file DataSheet_1.pdf]

Supplementary Material

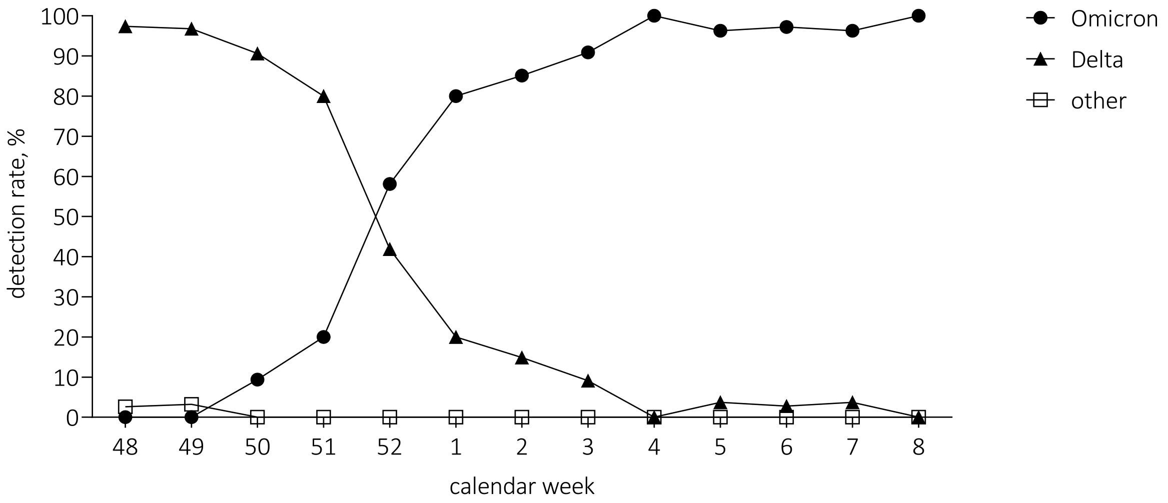

Supplementary Figure 1. Comparison of detection rates of different SARS-CoV-2 variants detected in patients and healthcare workers at the University Hospital Essen between week 48, 2021, and week 8, 2022. During week 52, 2021, Omicron (58.1 %) displaced Delta (41.9 %) as the most common variant. In week 8, 2022, only the Omicron variant (100.0 %) was detected.

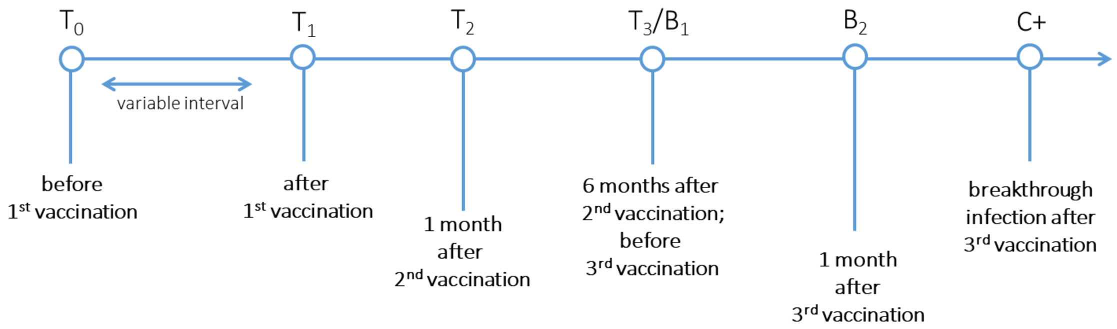

Supplementary Figure 2. Time points of blood/serum collection from study participants before and after vaccinations with either Moderna Biotech (mRNA-1273), BioNTech/Pfizer (BNT162b2), or AstraZeneca (AZD1222) vaccines. Vaccination scheme based upon recommendations of the national vaccine commission (STIKO).

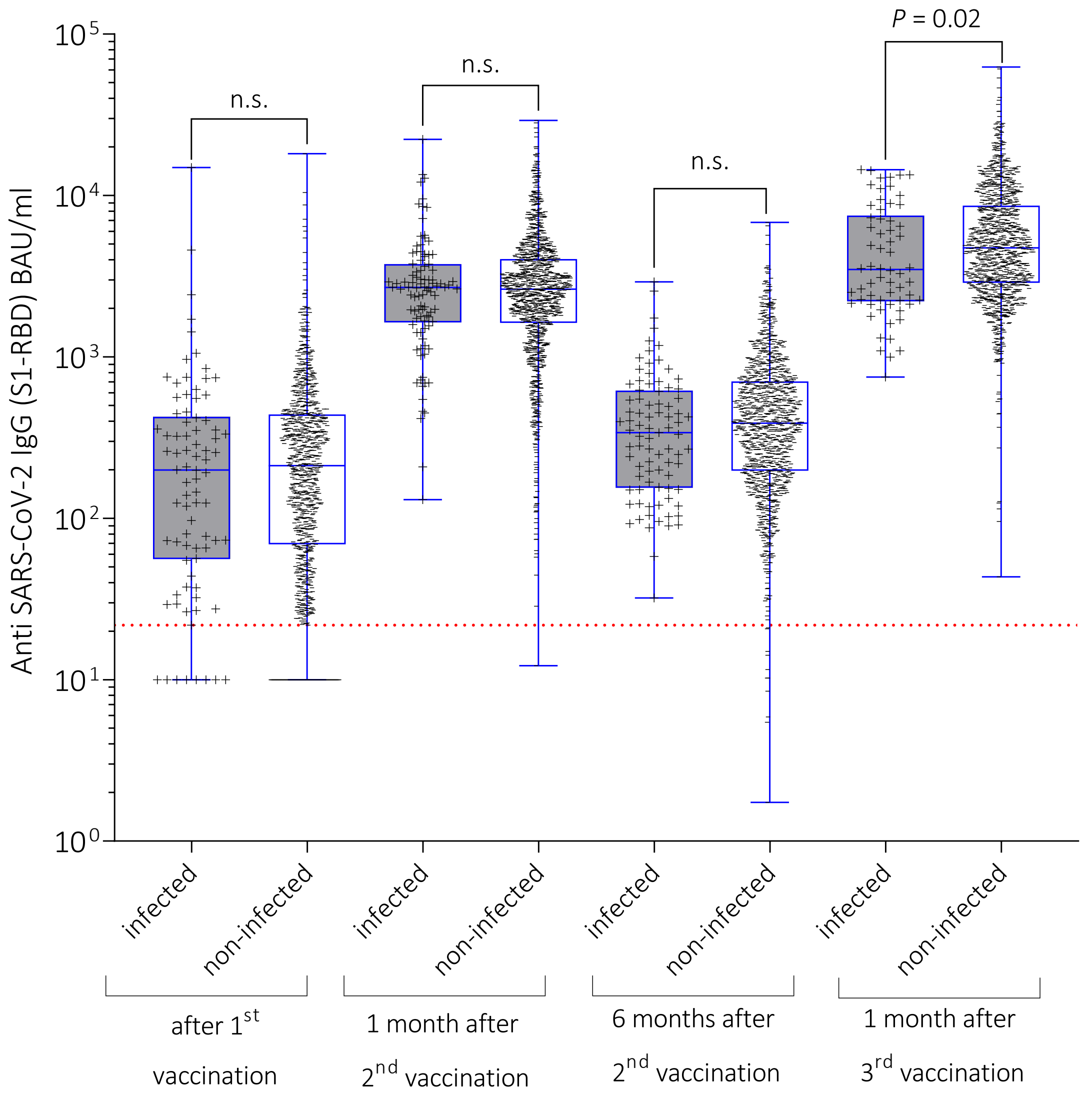

Supplementary Figure 3. Distribution of anti-spike antibody levels in SARS-CoV-2-positive (+, grey boxplots) and SARS-CoV-2-negative (-, white boxplots) study participants. The red dashed line indicates the detection limit for positivity (≥ 21.8 BAU/ml) of the test (SARS-CoV-2 S1 RBD IgG/sCOVG, Siemens Healthineers). Differences between anti-spike antibody titers in infected and non-infected individuals were analyzed by Mann-Whitney test, respectively. No differences in antibody titers were observed after first (199.1 BAU/ml vs 211.8 BAU/ml, $P = 0.49$), one month after second (2689.0 BAU/ml vs 2630.0 BAU/ml, $P = 0.79$) and six month after second vaccination (339.9 BAU/ml vs 388.9 BAU/ml, $P = 0.11$). Anti-spike antibody levels differed significantly one month after third vaccination (3477.0 vs 4733.0 BAU/ml, $P = 0.02$). Abbreviation: BAU/ml = binding antibody units per milliliter of serum; n.s. = not significant.

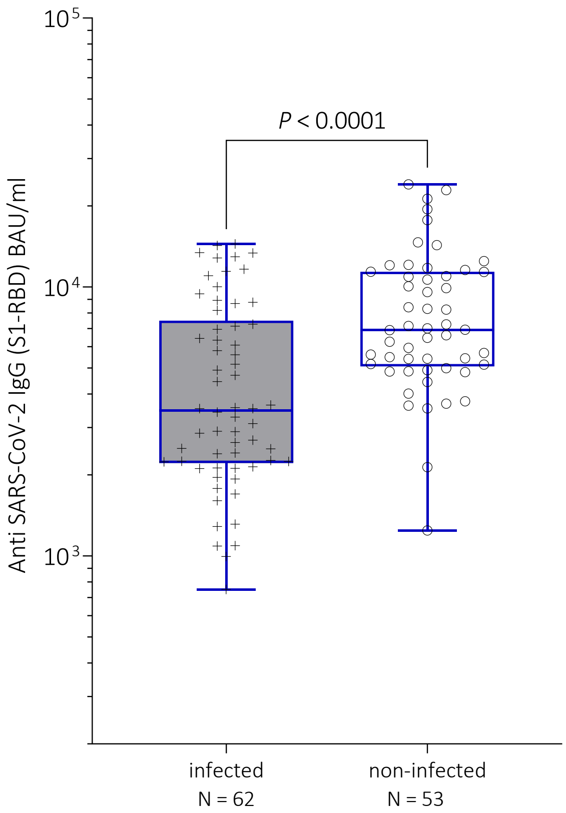

Supplementary Figure 4. Comparison of anti-spike antibody levels in breakthrough infection cohort (+, grey boxplots) and matched non-infected controls (o, white boxplots). Antibody levels were determined one month after the third vaccination (booster). Median values between infected (3477.0 BAU/ml) and non-infected (6935.0 BAU/ml) individuals differed significantly (Mann-Whitney test; $P < 0.0001$).

Supplementary Table 1. Demographics and vaccination scheme in subjects with and without breakthrough infection after third vaccination. Association of age, body mass index (BMI, kg/m^2) to breakthrough infection were estimated by Mann-Whitney test. *P*-value, odds ratio (OR) and 95 % confidence interval (CI) were calculated for association of sex, pre-existing conditions, smoking status, or vaccination scheme to SARS-CoV-2 vaccination breakthrough infection. Abbreviations: M = Moderna Biotech, mRNA-1273; B = BioNTech/Pfizer, BNT162b2; A = AstraZeneca, AZD1222.

	SARS-CoV-2-positive (N = 102)	SARS-COV-2-negative (N = 1289)	P-value, Odds ratio [95% Confidence Interval]
**Age (years)**	37.0 (19 - 63)	41.0 (18 - 72)	*P* = 0.004
**Sex**			
Male	26 (25.4 %)	290 (22.4 %)	*P* = 0.49, 1.18 [0.74 - 1.87]
Female	76 (74.5 %)	999 (77·5 %)	
**Pre-existing conditions**	29 (28.4 %)	333 (25.8 %)	*P* = 0.56, 1.14 [0.73 - 1.78]
**Smoker**	9 (8.8 %)	139 (10.8 %)	*P* = 0.54, 0.80 [0.40 - 1.62]
**Body mass index (kg/m^2)**	24.1 (17.7 - 43.6)	23.9 (16.7 - 56.8)	*P* = 0.25
**Vaccination scheme**			
M/M/B, or M	60 (58.8 %)	775 (60.1 %)	*P* = 0.79, 0.95 [0.63 - 1.43]
B/B/B, or M	24 (23.5 %)	334 (25.9 %)	*P* = 0.60, 0.88 [0.55 - 1.41]
A/A, B or M/B, or M	18 (17.6 %)	180 (13.9 %)	*P* = 0.31, 1.32 [0.77 - 2.25]
